# The effect of PRF (platelet-rich fibrin) inserted with a split-flap technique on soft tissue thickening and initial marginal bone loss around implants: results of a randomized, controlled clinical trial

**DOI:** 10.1186/s40729-016-0044-4

**Published:** 2016-05-04

**Authors:** Julia Hehn, Thomas Schwenk, Markus Striegel, Markus Schlee

**Affiliations:** 1 Periodontology, Edel & Weiß Clinic, Ludwigsplatz 1a, 90403 Nuremberg, Germany; 2 Esthetic Dentistry, Edel & Weiß Clinic, Nuremberg, Germany; 3 Private Clinic for Periodontology, Forchheim and Department of Maxillofacial Surgery, Goethe University, Frankfurt, Germany

**Keywords:** Soft tissue augmentation, PRF (platelet-rich fibrin), Bone level, Initial bone loss, Implants, Mucosal tissue thickness, RCT, Split-flap

## Abstract

**Background:**

Previous studies have shown that adequate thickness or initial augmentation of soft tissue has a positive effect on the stability of peri-implant bone. This randomized, controlled trial aimed to evaluate the influence of augmenting soft tissue with platelet-rich fibrin (PRF) on crestal bone and soft tissue around implants.

**Methods:**

After randomization, 31 fully threaded titanium implants were inserted in 31 patients (16 men and 15 women) in the lower mandible using a split-flap technique. In the test group (10 patients), mucosa was treated with a PRF membrane. In the control group (21 patients), implantation was realized without soft tissue augmentation. Tissue thickness was measured at point of implant insertion (baseline) and at time of reentry after 3 months. Standardized digital radiographs were obtained for evaluation at time of implant placement, reentry after 3 months and at a 6-month follow-up. Data was analyzed by an independent examiner.

**Results:**

After 6 months, all 31 implants were osteointegrated. Soft tissue augmentation with PRF led to a significant tissue loss. In the test group, the crestal tissue thickness dropped from 2.20 mm ± 0.48 SD at baseline to 0.9 mm ± 1.02 SD at reentry, whereas crestal mucosa in the control group showed higher stability (2.64 mm ± 0.48 SD at baseline to 2.62 mm ± 0.61 SD at reentry). For ethical reasons, the test group was terminated after 10 cases, and the remaining cases were finished within the control group. In the test group, radiographic evaluation showed a mean bone loss of 0.77 mm ± 0.42 SD/0.57 mm ± 0.44 SD (defect depth/defect width) on the mesial side and 0.82 mm ± 0.42 SD/0.62 mm ± 0.36 SD (defect depth/defect width) on the distal side. In the control group, a mean bone loss of 0.72 mm ± 0.61 SD/0.51 mm ± 0.48 mm (defect depth/defect width) on the mesial and 0.82 mm ± 0.77 SD/ 0.57 mm ± 0.58 SD (defect depth /defect width) on the distal side was measured.

**Conclusions:**

Within the limits of this study and the early determination of the test group, this study concludes that soft tissue augmentation with PRF performed with a split-flap technique cannot be recommended for thickening thin mucosa. Further studies focusing on different techniques and longer follow-ups are needed to evaluate whether PRF is suitable for soft tissue thickening.

## Background

The initial bone modeling around implants within the first year after insertion presents a challenging topic in current research. Previous studies have shown that this process is characterized by a remodeling of the horizontal and vertical bone dimension with a range of 0.7 to 3 mm within the first year [1].

First attempts to reduce this loss of tissue focused on changes of implant shapes, implant surfaces, implant position, and abutment design. The concept of “platform switching” seemed to be the most promising step in this process. Hürzeler et al. and Vela-Nebot et al. reported a reduced loss of bone substance with platform-switched abutments when compared to regular abutments after a 6-month loading. Cochran et al. and Canullo et al. support this thesis, whereas Becker et al. could not report any histological differences between platform-switched and regular abutments [2–6]. However, initial bone modeling could not be fully avoided in the abovementioned studies.

A second approach to reduce bone loss is the altering of peri-implant soft tissue. Berglundh et al. showed that the peri-implant mucosa has many features in common with the gingiva around natural teeth [7]. Around implants, a biological constant is formed comparable to the biological width, characterized by a thick epithelial layer of 2 mm and a suprabony connective tissue layer of 1 mm. Abrahamsson published similar results, showing that the dentogingival complex around implants ranges from 3.5 mm to 4 mm [8]. Lindhe et al. reported that thinning or destruction of this tissue thickness leads inevitably to peri-implant bone resorption [9]. This implies that the existence of a minimum of peri-implant mucosa thickness is crucial for the long-term stability of the bone level.

Linkevicius et al. showed that the initial tissue thickness influences crestal bone changes around implants [10, 11]. After 6 months, the group of patients with thin tissue (up to 2 mm) had a mean bone loss of 1.35 mm+/−0.33 SD, whereas patients with normal tissue or thick tissue (3.1 mm and more) showed significantly less bone loss (0.32 mm+/−0.44 SD and 0.12 mm+/−0.52 SD, respectively) [10].

As a consequence, recent research focused on soft tissue augmentation of thin gingiva types prior to or simultaneous to implant insertion. Wiesner et al. published a significant gain of soft tissue by thickening the gingiva with a connective soft tissue graft harvested from the palate [12]. Soft tissues at augmented sites were 1.3 mm thicker than on control sites and had a better pink esthetic score. However, this technically sensitive procedure did not lead to less peri-implant bone loss (0.8 mm in the grafted group, 0.6 mm in the non-grafted group after 1-year loading).

Lai et al. and Linkevicius et al. reported a successful vertical augmentation of soft tissue by using an acellular dermal matrix membrane [13, 14]. Furthermore, Linkevicius showed that thickening with an allogenic membrane resulted in significantly reduced initial bone loss (0.21-mm bone loss in the augmented group, 1.17-mm bone loss in the control group after 1-year follow-up) [14].

Another attempt to influence the peri-implant bone structures is the use of platelet-rich fibrin (PRF). This second-generation platelet concentrate described by Choukroun et al. is a fibrin matrix enriched with cytokines, circulating progenitor cells, and growth factors which can be used as a resorbable membrane in surgery. Studies show a constant release of growth factors such as PDGF (platelet-derived growth factor) or TGF-b (transforming growth factor) for at least 1 week [15, 16] up to 28 days [17] and proved its accelerating effect on the healing process. The application of PRF has been tested in various disciplines of dentistry so far. However, no data of vertical soft tissue augmentation with PRF has yet been published.

The aim of this randomized, controlled clinical trial was to evaluate whether the use of PRF is suitable for soft tissue thickening of the peri-implant mucosa and to assess the marginal bone level changes.

## Methods

### Patient selection

Patients aged 18+ who required an implant in the posterior mandible were eligible for this study.

Exclusion criteria were the following:

- general contraindications to implant surgery
- insufficient oral hygiene and periodontitis
- patients with a history of severe periodontitis
- bone augmentation required
- smokers
- substance abuse
- uncontrolled diabetes
- severe cardiovascular problems
- treated or under treatment with intravenous amino-bisphosphonates
- pregnant or lactating.

The study was conducted in accordance with the standards of the Declaration of Helsinki of 1983 and was approved by the ethics committee of the FEKI (Freiburger Ethik-Kommission International, Feki Code: 014/1210). All 40 recruited patients were informed about the design and aim of this study, and written consent was obtained. The randomization to control group (*n* = 20) and test group (*n* = 20) was achieved using a sealed envelope system at time of surgery.

### Clinical procedure

All patients had to undergo a professional dental hygiene treatment in advance. One hour prior to surgery, patients were given an antibiotic single shot prophylaxis (600 mg clindamycin). PRF was obtained from each patient of test and control groups and treated according to the PRF protocol with an IntraSpin™ table centrifuge and collection kits provided by Botiss (Zossen, Germany).

After anesthesia with Ultracain® DS-forte (articaine + adrenaline 1:100,000), crestal, lingual, and buccal tissue thickness was tested using an endodontic micro-spreader (Spreader ISO 30, Dentsply Maillefer®) with a silicon stop (illustration 1). The instrument part penetrating the soft tissue was measured with an endodontic longimeter. Measured data was rounded off to the nearest half millimeter (mm).

**Illustration 1:**
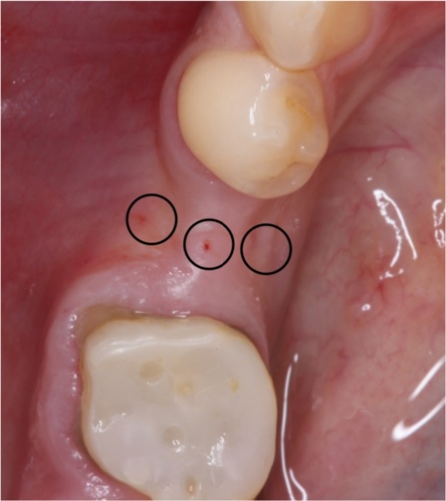
Measurement of tissue thickness with an endodontic micro-opener

The initial preparation of the split-flap was carried out the same way in test and control groups using microsurgical instruments. After a crestal incision with a microsurgical blade (SM69, Swann Morton LTD®, Sheffield, England), the split-thickness flap was sharply prepared by elevating the area of the single tooth gap to the middle of the adjacent teeth. The periosteum was split to receive a tension-free adaption of the flaps (illustration 2). Fully threaded titanium implants (Nobel Speedy Replace®, Nobel Biocare, Zurich, Switzerland) were inserted at bone level with primary stability. The implants varied in diameter (narrow platform 3.5 mm, regular platform 4.0 mm, wide platform 5.0 mm) and in length (10 mm, 11.5 mm, 13 mm) (illustrations 3, 4, and 5). For the further procedure, patients were now randomized by a dental assistant using a sealed envelope system. In the test group, the tissue was augmented with a PRF membrane using a double-layered technique. In the control group, the implant treatment was realized without mucosa thickening (illustrations 6 and 7). Flaps were sutured with a non-absorbable polyvinylidene fluoride suture (Seralene®, Serag Wiessner, Naila, Germany) (illustration 8).

**Illustration 2:**
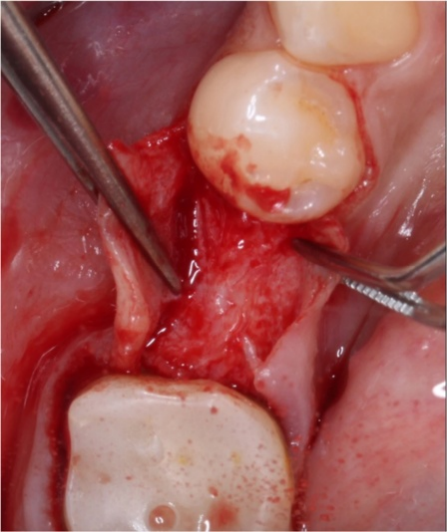
Crestal incision and preparation of a split-flap

**Illustration 3:**
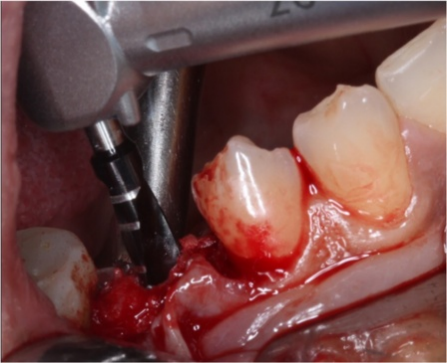
Insertion of the implant

**Illustration 4:**
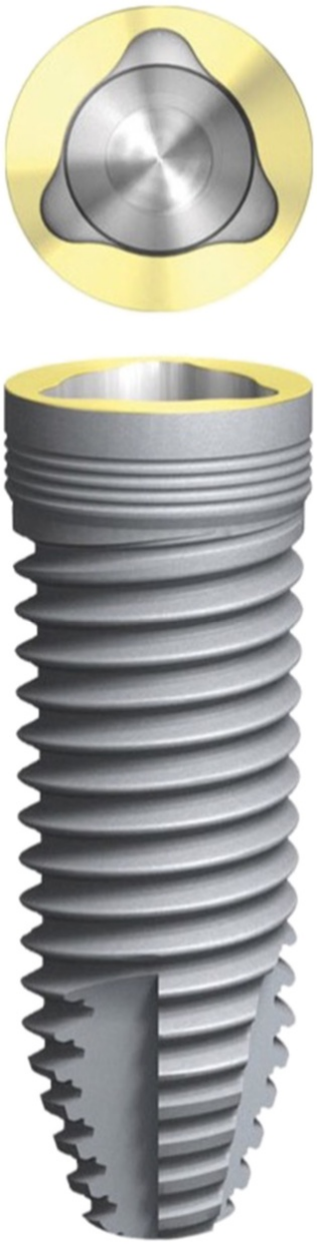
NobelSpeedy Replace® (source: https://www.nobelbiocare.com/de/de/home/products-and-solutions/implant-systems/nobelspeedy.html)

**Illustration 5:**
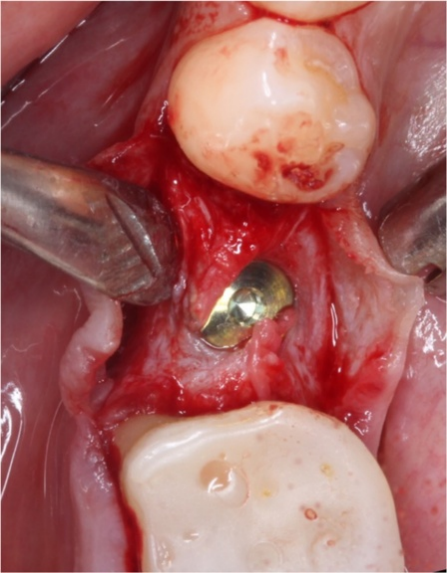
Implant placed with a split-flap technique

**Illustration 6:**
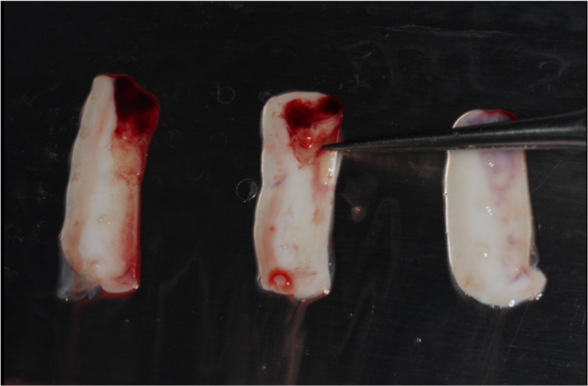
PRF membrane made by centrifugating and pressing the patient’s blood

**Illustration 7:**
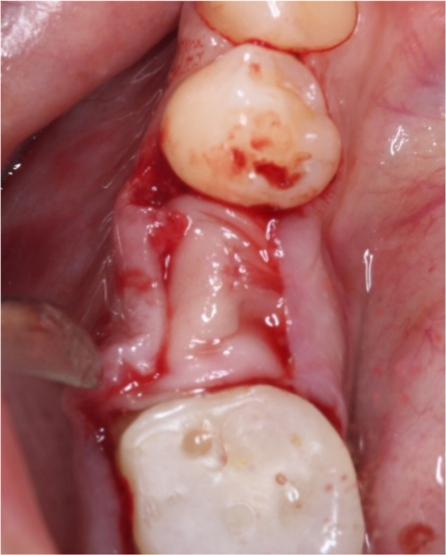
Insertion of PRF membranes in a double-layered technique for tissue augmentation

**Illustration 8:**
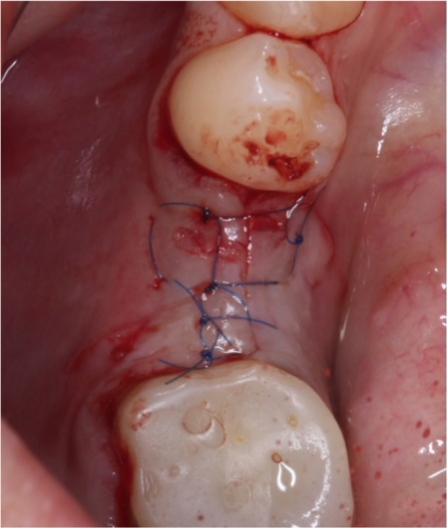
Fixation of the flap with Seralene® 6.0

After the implantation, standardized digital X-rays were taken with parallel technique (baseline) (illustration 9). For each patient, an individual customized digital film holder was fabricated to ensure a reproducible radiographic analysis. Patients were instructed to avoid chewing hard nutrition in the treated area and to use chlorhexidine mouthwash and a soft brush twice a day for the first 2 weeks. Sutures were removed after 7 to 10 days.

**Illustration 9:**
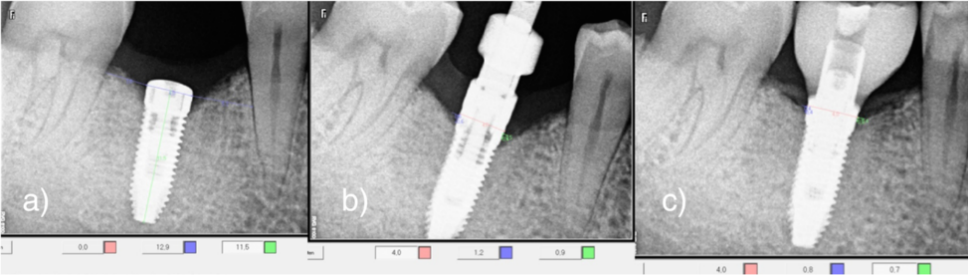
**a**–**c** Radiographic control **a** at time of implant placement (**a**), 3 months post-operative (**b**), and 6 months post-operative (**c**)

Three months later, a second measurement of crestal, buccal, and lingual tissue thickness and intraoral radiographs were carried out as mentioned above. A small mid-crestal incision was made and cover screws were replaced by healing abutments. Due to the minimal invasive reentry procedure, no sutures were needed. Within the following 2 weeks, one-piece, screwed, full ceramic crowns were inserted as definite restorations (IPS Emax®, Ivoclar Vivadent, Schaan, Liechtenstein; composite: Multilink Implant®, Ivoclar Vivadent, Schaan, Liechtenstein) (illustration 10). In a 6-month follow-up from baseline, patients were recalled for maintenance and another digital X-ray was taken.

**Illustration 10:**
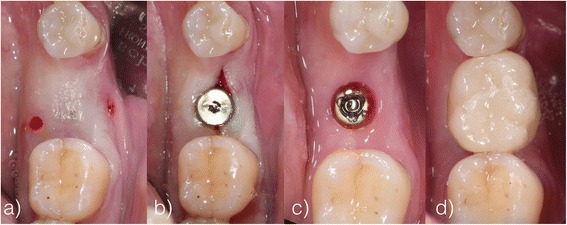
**a**–**d** Second measuring, reentry, and insertion of a screwed, full ceramic crown

### Radiographic assessments

The evaluation of the peri-implant bone remodeling was carried out by an independent examiner, who was not involved in the surgical process. Bone loss at the mesial and distal side of each implant was measured with an image analysis software (Kodak dental imaging software, version 6.13.0, 2013). A calibration of length measurement was performed in every radiograph to avoid radiographic distortion (reference value - implant length and width).

### Statistical analysis

Data of 31 patients (10 patients of test group and 21 patients of control group) were analyzed with STATISTICA (version 9.1, StatSoft, Inc., Tulsa, USA) and BiAS (version 10.11, Epsilon, Frankfurt, Germany). No data points were missing.

The analysis focused on the following aspects:

- Comparison of tissue thickness (crestal, buccal, lingual) at baseline and 3-month data in test and control groups
- Comparison of tissue gain/loss between test and control groups
- Comparison of mesial and distal bone level at baseline, 3-month and 6-month data in test and control groups
- Comparison of bone level alterations between test and control groups.

Data were expressed as means ± standard deviation. Comparisons were made using the Wilcoxon test, the Mann–Whitney *U* test, and the multiple comparisons test by Schaich-Hamerle (*p* = 0.05).

## Results

At time of surgery, the patients ranged in age from 33 to 79 years (mean age 53.8 years).

The first surgeries for implant placement in the test group were carried out as described above. Two layers of a PRF matrix were placed on top of the implant. Though surgical flaps were all sutured completely free of tension, a post-operative dehiscence above the implant could be observed in all test patients within the first week. This process resulted in a complete loss of mucosal and augmented tissues above the implant. The open areas were healed by secondary intention (illustration 11). The wounds in the control group healed uneventfully by primary intention; no dehiscences occurred.

**Illustration 11:**
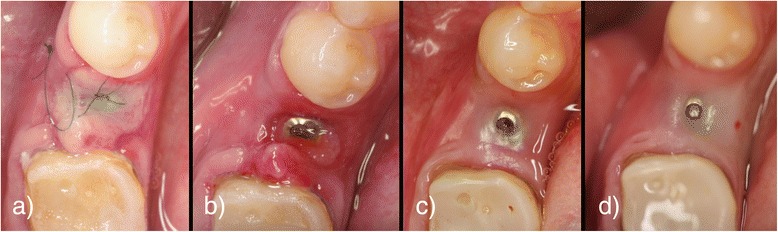
**a**–**d** Post-operative healing process at 3 days post-operative (**a**), 1 week post-operative (**b**), 1 month post-operative (**c**), and 3 months post-operative (**d**)

Due to ethical reasons, the test group had to be terminated after 10 patients. The already recruited, remaining patients were consequently all added to the control group. Finally, the study was finished with a total number of 31 consecutive patients (16 males and 15 females, 10 patients in the test group, 21 in the control group).

No drop-out occurred within the 6 months. All implants were clinically osseointegrated and stable and showed no sign of infection.

### Mucosa thickness

Crestal mucosa thickness in test group dropped from 2.20 mm ± 0.48 SD at baseline to 0.90 mm ± 1.02 SD at reentry. This loss was statistically significant. Buccal mucosa thickness was 1.85 mm ± 0.41 SD at baseline and 2.15 mm ± 0.78 SD at 3-month follow-up, and lingual thickness started from 1.55 mm ± 0.44 SD and resulted in 1.80 mm ± 0.63 SD. Buccal and lingual data did not reach statistical significance. Within the limited data provided by the early termination of the study, it can be stated that PRF under the condition of a split-flap design failed to improve the thickness of the mucosa.

**Fig. 1:**
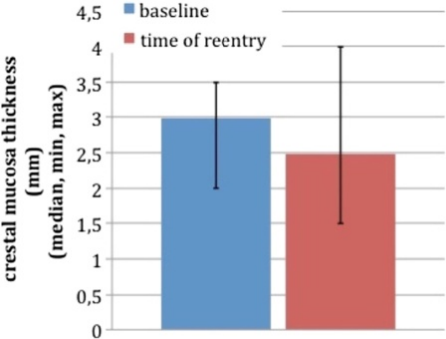
Control group (no PRF augmentation)

**Fig. 2:**
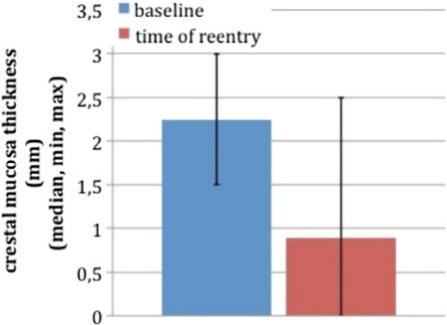
Test group (PRF augmentation)

In the control group, crestal mucosa thickness decreased from 2.64 mm ± 0.48 SD at baseline to 2.62 mm ± 0.61 SD at 3-month follow-up. Buccal and lingual mucosa was 2.29 mm ± 0.54 SD resp. 1.62 mm ± 0.55 SD at baseline and dropped to 2.36 mm ± 0.48 SD resp. 1.86 mm ± 0.53 SD. The differences of all three measuring points were not statistically significant (Figs. 1 and 2).

### Marginal bone level

**Fig. 3:**
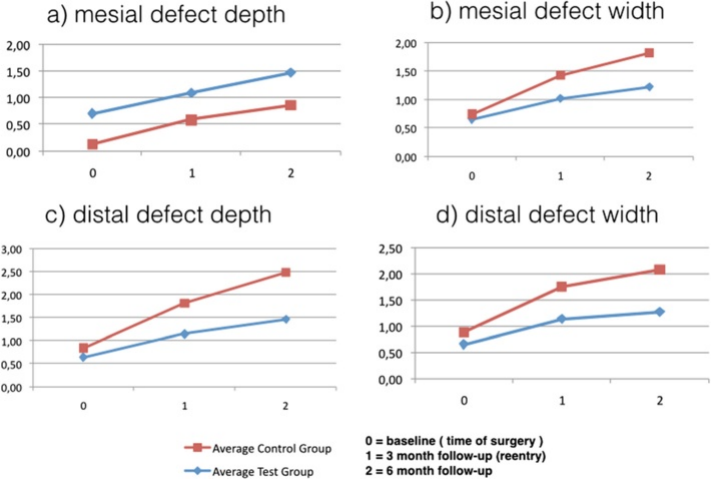
Peri-implant bone level at baseline, 3 months post-operative, and 6 months post-operative. **a** Mesial defect depth. **b** Mesial defect width. **c** Distal defect depth. **d** Distal defect width

The mean marginal bone level alterations are displayed in Fig. 3.

Six months after surgery, both groups showed a statistically significant peri-implant bone loss.

#### Radiographic evaluation of bone level in the test group

The mesial resp. distal defect depth was 0.70 mm ± 0.72 SD resp. 0.64 mm ± 0.66 SD at baseline. Six months later, the marginal bone defect increased to 1.47 mm ± 0.65 SD on the mesial side resp. 1.46 mm ± 0.54 SD on the distal side. The corresponding defect width changed from 0.7 mm ± 0.72 SD to 1.22 mm ± 0.57 SD (mesially) and from 0.65 mm ± 0.64 SD to 1.27 mm ± 0.55 SD (distally).

#### Radiographic evaluation of bone level in the control group

In the control group, defect depth changed from 0.13 mm ± 0.26 SD to 0.86 mm ± 0.52 SD (mesially) and from 0.2 mm ± 0.37 SD to 1.02 mm ± 0.62 SD (distally). Defect width increased from 0.09 mm ± 0.17 SD to 0.60 mm ± 0.47 SD (mesially) and from 0.24 mm ± 0.42 SD to 0.81 mm ± 0.44 SD (distally).

#### Comparison of differences in bone loss between test and control groups

The test group showed a mean bone loss of 0.77 mm ± 0.42 SD/0.57 mm ± 0.44 SD (defect depth/defect width) on the mesial side and 0.82 mm ± 0.42 SD/0.62 mm ± 0.36 SD (defect depth/defect width) on the distal side.

In the control group, a mean bone loss of 0.72 mm ± 0.61 SD/0.51 mm ± 0.48 mm (defect depth/defect width) on the mesial side and 0.82 mm ± 0.77 SD/0.57 mm ± 0.58 SD (defect depth/defect width) on the distal side was measured.

However, the comparison of the differences in bone loss showed no significance between the control and test groups (all *p* > 0.05, Mann–Whitney *U* test).

## Discussion

In this study over a period of 6 months, it could be demonstrated that mucosal tissue thickening above implants with PRF led to reduced tissue thickness when performed in a split-flap technique.

The initial post-operative dehiscence and the associated complete loss of mucosal and augmented tissue above the implant were observed in all test patients.

PRF is supposed to be a good healing aid in various aspects of dentistry [18]. Choukron and his associates introduced this technique to implant dentistry to improve bone healing [15]. According to his studies, the natural fibrin framework protects growth factors from proteolysis, so they can stay active for a longer period (up to 28 days [17]). This leads to an effective neovascularization and an accelerated wound closing with less post-operative infections [16, 19]. Though PRF has been tested successfully in surgical procedures with reference to hard tissue augmentation (sinus lift, socket preservation) [20, 21] and in the field of periodontal regeneration [22], publications of PRF usage in combination with soft tissue augmentation are rare and allow no real conclusion so far.

The first assumption that comes up is whether the higher tension on the flaps caused by the additional volume of the PRF membranes was adequately compensated. This can be clearly affirmed. Split flaps were expanded widely until the middle of the adjacent teeth. In addition to that, a very sensitive suture material was used to secure an adaption completely free of tension (illustration 8). The essential point that needs to be critically reviewed in this study is the insertion of PRF in combination with a split-flap technique. The bilayered insertion of PRF allows a better nutrition of the augmentation material itself and avoids higher peri-implant bone loss due to trauma and infection of the periosteum [22]. However, this highly technique-sensitive procedure requires much experience. Moreover, the initial thin mucosa is split into two extremely thin layers. A sufficient nutrition of the flap requires a minimum flap thickness of 0.8–1.2 mm [23, 24]. Since the blood supply of these flaps with reduced thickness cannot be provided only from the lateral, additional nutrition from the periosteum and the bone is necessary to maintain a livid flap. This may explain the punctual dehiscence above the implant itself, which occurred in all 10 test patients. According to the authors’ observations during this study, the nutrition problem coming up with the split-flap technique seems to be the crucial factor for the poor results with reference to soft tissue thickening.

With respect to marginal bone loss, it could be shown that there were no significant differences when comparing dimension of bone loss between test and control groups. The study of present research data shows, to the best knowledge of the authors, no other RCTs about tissue thickening with PRF and peri-implant bone loss. However, several studies focused on mucosa thickening with tissue grafts and assessed the marginal bone loss around implants. Hehn et al. and Wiesner et al. achieved a stable mucosa thickening by augmenting the initial situation with a tissue graft taken from the palate [12, 25]. Whereas Hehn could show a reduced bone loss after 12 months, Wiesner’s results show no difference in bone loss between test and control sides.

A second technique is the use of membranes. Lai et al. postulated a successful, long-term stable augmentation by using an acellular dermal matrix (ADM group increased by 3.10+/−0.64 mm at 12 weeks, control group increased by 0.30+/−0.50 mm) [13]. Puisys et al. focusing on the coherence of membrane tissue augmentation and peri-implant bone loss show similar results. They came to the conclusion that initial soft tissue augmentation with an allogenic collagen matrix resulted in a long-term stable thickened mucosa. Furthermore, the bone loss around implants was significantly less when implants were placed in naturally thick tissue than in thin tissue. Bone loss 1 year after surgery around implants with a thin mucosa was 1.22+/−0.08 mm bone loss mesially and 1.14+/−0.07 mm distally. Around implants placed in a thick mucosa, it was 0.24+/−0.06 mm mesially and 0.19+/−0.06 mm distally. The attempt to thicken thin mucosa with an allogenic membrane resulted in highly reduced bone loss (0.22+/−0.06 mm mesially and 0.20+/−0.06 mm distally after 1 year) [11].

One limitation of this study is the standardized radiographic evaluation. This technique allows information about the peri-implant bone only on the mesial and distal but not on the buccal and lingual side. However, this fact is limiting most present studies focusing on the peri-implant bone level [5, 26]. Yet, the main limitation of this study is the small number of patients. For this reason, these findings cannot be generalized to soft tissue augmentation with PRF on this study alone.

## Conclusions

Soft tissue augmentation with PRF using a split-flap technique cannot be recommended to alter thin gingiva types. Future experimental and clinical studies will be necessary to evaluate whether augmentation with PRF is suitable for mucosa thickening.

## Abbreviations

PDGF: platelet-derived growth factor
PRF: platelet-rich fibrin
SD: standard deviation
TGF-b: transforming growth factor-b
